# Recent advances in the treatment of Ebola disease: A brief overview

**DOI:** 10.1371/journal.ppat.1012038

**Published:** 2024-03-15

**Authors:** L’Emir Wassim El Ayoubi, Omar Mahmoud, Johnny Zakhour, Souha S. Kanj

**Affiliations:** 1 Division of Infectious Diseases, Department of Internal Medicine, Faculty of Medicine, American University of Beirut, Beirut, Lebanon; 2 Division of Public Health, Infectious Diseases and Occupational Medicine, Department of Medicine, Mayo Clinic, Rochester, Minnesota, United States of America; 3 Internal Medicine Department, Henry Ford Hospital, Detroit, Michigan, United States of America; 4 Center for Infectious Diseases Research, American University of Beirut, Beirut, Lebanon; University of Iowa, UNITED STATES

## Abstract

Ebola disease (EBOD) remains a significant and ongoing threat to African countries, characterized by a mortality rate of 25% to 90% in patients with high viral load and significant transmissibility. The most recent outbreak, reported in Uganda in September 2022, was declared officially over in January 2023. However, it was caused by the Sudan Ebola virus (SUDV), a culprit species not previously reported for a decade. Since its discovery in 1976, the management of EBOD has primarily relied on supportive care. Following the devastating outbreak in West Africa from 2014 to 2016 secondary to the Zaire Ebola virus (EBOV), where over 28,000 lives were lost, dedicated efforts to find effective therapeutic agents have resulted in considerable progress in treating and preventing disease secondary to EBOV. Notably, 2 monoclonal antibodies—Ebanga and a cocktail of monoclonal antibodies, called Inmazeb—received Food and Drug Administration (FDA) approval in 2020. Additionally, multiple vaccines have been approved for EBOD prevention by various regulatory bodies, with Ervebo, a recombinant vesicular stomatitis virus-vectored vaccine against EBOV being the first vaccine to receive approval by the FDA in 2019. This review covers the key signs and symptoms of EBOD, its modes of transmission, and the principles guiding supportive care. Furthermore, it explores recent advancements in treating and preventing EBOD, highlighting the unique properties of each therapeutic agent and the ongoing progress in discovering new treatments.

## Introduction

Ebola disease (EBOD) is a rare but often fatal disease that affects humans and other primates. It is caused by viruses from the *Ebolavirus* genus, filoviruses with a linear single negative-stranded ribonucleic acid (RNA), with 2 species, the Zaire Ebola virus (EBOV) and the Sudan Ebola virus (SUDV) accounting for most disease burden in humans [1]. EBOD was first identified in 1976 during 2 simultaneous outbreaks in Sudan and the Democratic Republic of Congo (DRC) [2]. Subsequently, 39 outbreaks of EBOD have occurred in Africa, as documented by the Center for Disease Control and Prevention (CDC), with multiple outbreaks reported in countries outside of the African continent throughout the years [3]. The largest outbreak to date was reported in West Africa between 2014 and 2016, affecting more than 28,000 people with a case fatality rate of 39.5% [2]. EBOD remains an ongoing infectious health concern with a significant potential to cause epidemics. In this review, we will explore recent advances in the treatment of EBOD. It should be noted that we will refer to diseases secondary to any *Ebolavirus* species as “EBOD,” whereas “Ebola Virus Disease” (EVD) will refer specifically to disease secondary to EBOV. This terminology is in accordance with the expert consensus accepted by the 11th International Classification of Diseases for filoviruses (ICD-11) in 2019 [4].

## 1. What are the presenting signs and symptoms of EBOD?

EBOD initially presents with nonspecific symptoms such as fever, headache, myalgia, and gastrointestinal disturbances, typically appearing between 2 to 21 days following exposure [5]. However, it can rapidly progress to multi-organ failure, mostly during the second week of illness [6,7]. Although early reports mentioned severe hemorrhage, it was not reported in more recent studies, prompting the shift in nomenclature from hemorrhagic fever to EBOD [8].

The mortality rate ranges between 50% and 90% [7]. While a shorter symptom duration before admission, lower baseline serum creatinine and aminotransferase levels, and a lower viral load at admission were each associated with improved survival [9], a longer duration of symptoms before treatment increased the odds of death by 11% for each day of delayed therapy [9].

## 2. How is the virus transmitted, and how to prevent its spread?

EBOD is transmitted to humans through direct contact with infected animals, such as fruit bats, or contact with the bodily fluids of infected individuals (blood, urine, sweat, saliva, breast milk, and semen…) [2].

Prevention and control measures include identifying and isolating infected individuals, tracing contacts, and promoting public awareness about the disease [10]. Organizations with extensive experience in the management of EBOD such as the World Health Organization (WHO) have issued infection prevention protocols to ensure the safety of healthcare workers and the prevention of transmission. These include adopting standard, contact, and droplet precautions, the use of personal protective equipment (PPE) that adequately covers the body, such as Tyvek suits to avoid contact with the bodily fluids of infected patients, in addition to the use of a powered air purifying respirator (PAPR) or, although less likely, N95 masks when PAPRs are not available [6,10,11].

## 3. What are the essential components of supportive care?

Supportive care and treatment of complications are the cornerstones of the treatment of EBOD [5]. This includes maintaining hydration, blood pressure monitoring, and nutritional support as well as symptomatic relief (analgesics, antipyretics…) [5]. Outpatient management should be attempted early if possible and if oral intake is tolerated, to reduce the strain on healthcare systems [6].

Intensive care unit admission may be required in up to 50% of admitted patients with EBOD for close monitoring, respiratory support, and management of severe complications [12]. Patients should be monitored for signs of dehydration and metabolic disturbances with careful monitoring of vital signs and urine output [6]. Correcting electrolyte disturbances is essential as serum potassium and acid-base disturbances have been associated with increased mortality in EBOD patients [6]. Anti-emetics and anti-diarrheal agents should be used to prevent intravascular depletion and maintain adequate renal perfusion. Additionally, when available, renal replacement therapy should be offered, when indicated [10]. Patients with EBOD may also require respiratory support, as 70% of the patients treated in Europe and the United States (US) required supplemental oxygen, and 47% of them had respiratory failure [13].

Cardiovascular complications should also be addressed promptly [14]. Intravenous vasopressors or inotropic agents should be quickly initiated in patients with severe hypotension that is unresponsive to volume resuscitation [10]. Signs of hemorrhage should be closely monitored. Management of thrombocytopenia and coagulation disorders can include vitamin K, tranexamic acid, and transfusion of blood products when indicated [10].

Unfortunately, some of these supportive measures including sometimes adequate PPEs are lacking in some areas suffering from outbreaks [15].

## 4. Is there any role for antimicrobial therapy in the treatment of EBOD?

Most expert opinions recommend empirical broad-spectrum antibiotics in critically ill EBOD patients since reports have indicated a higher susceptibility to bacterial infection [5,6,16]. This is due to an induced immune dysregulation by ebolaviruses, including the virus’s initial replication in macrophages, the inhibition of interferon-mediated antiviral responses, and cytokine-induced lymphopenia [1]. The main secondary infections of concern are malaria and bacterial infections. However, comprehensive reports of the actual incidence and epidemiological profile of secondary bacterial infections in patients with EBOD are yet to be published [17], with evidence on empiric broad-spectrum antibiotic coverage being mainly based on case reports and expert opinion [6,18].

The rationale for empiric antibiotics is to address potential undiagnosed bacterial infections in critically ill EBOD patients, especially in settings with limited diagnostic capabilities. For instance, gram-positive coverage may be needed in patients with central catheters [10]. Additionally, some reports stress the utility of Gram-negative coverage due to reported cases of EBOD-associated Gram-negative bacteremia [18], which was mainly attributed to the increased risk of bacterial translocation from the gut [1].

However, excessive antibiotic use has been reported in patients with EBOD [13,19], and recent evidence suggests that rates of bacterial coinfections and superinfections are not significantly elevated in patients with EBOD compared to patients without EBOD [17,20]. Given the increasing concern over antimicrobial resistance, especially in low- and middle-income countries (LMICs) where EBOD is prevalent, it is imperative to limit empirical antibiotic use unless there is a clinical suspicion of bacterial infection [6]. Empirical antimicrobials should be reevaluated after 48 h of initiation and should be discontinued if deemed appropriate as mentioned in the WHO’s guidelines for supportive care of patients with EBOD [21].

As for *Plasmodium* spp., it is advised to empirically treat for coinfection both in suspected or confirmed EBOD and critically ill EBOD patients from malaria-endemic regions using artemisinin-based therapies when no diagnostic tests are available, with treatment to be reassessed after means of diagnosis is available or the course of empirical therapy is completed [1,10].

## 5. What are the available targeted therapies against EBOD?

Most of the therapeutic options studied and available mainly target EVD, EBOD secondary to EBOV. Currently, there are 2 Food and Drug Administration (FDA)-approved agents for the treatment of EVD: atoltivimab-maftivimab-odesivimab (Inmazeb) and ansuvimab (Ebanga), both receiving FDA approval in October and December 2020, respectively [22]. This breakthrough marked a shift in the 44-year era of supportive treatment since the initial outbreaks in DRC and Sudan in 1976 [16,22].

Ebanga, also known as MAb114, is a monoclonal antibody (mAb) derived from memory B cells of 1995 Kikwit EVD survivors [22]. Ebanga targets the EBOV surface glycoprotein (GP), impeding virus entry by neutralizing the virus and initiating antibody-dependent cellular cytotoxicity (ADCC) [1,22]. It is administered through a single 60-min infusion (50 mg/kg) and is recommended in all age groups, including pregnant and breastfeeding women [16,22].

Inmazeb, or REGN-EB3, is a combination of 3 human immunoglobulins initially sourced from mice immunized with DNA expressing the EBOV GP gene. Each mAb targets a distinct GP epitope, collectively neutralizing the virus and triggering ADCC and phagocytosis. It is also administered via a single infusion (150 mg/kg) and is proven to be as safe as Ebanga [16,22].

The efficacy of Ebanga and Inmazeb has been demonstrated in the PALM trial, conducted during the 2018 to 2020 North-Kivu and Ituri epidemics [22]. This randomized clinical trial aimed to compare 3 new investigational products (Inmazeb, Ebanga, and remdesivir) to ZMAPP, which was considered the control arm given its promising results in the PREVAIL II trial, with a primary endpoint of mortality at 28 days. It enrolled a total of 681 patients with laboratory-confirmed EVD, who were randomly assigned in a 1:1:1:1 ratio to one of the 4 investigational products, including remdesivir and ZMAPP. The use of these products was authorized under the Monitored Emergency Use of Unregistered Interventions (MEURI) protocol, which justifies the use of investigational products to advance the search for new treatment options for EVD. This study successfully proved that Inmazeb and Ebanga improve survival with few side effects, surpassing ZMAPP and remdesivir in terms of mortality. However, it showed no evidence of superiority between these 2 mAb products. In response, the WHO guidelines conditionally recommended against ZMAPP and remdesivir [16].

ZMAPP, a cocktail of mAbs targeting EBOV’s GP, was the first mAb product investigated for the treatment of EVD as part of the MEURI protocol. It showed promising results in cell culture studies, however, failed to meet the prespecified statistical threshold for efficacy when evaluated in a phase II clinical trial [23].

Various polymerase inhibitors (PIs) other than remdesivir have also been investigated. Brincidofovir, despite the initial promise in cell culture studies [24], had the phase II clinical trial terminated in 2015 after the manufacturer decided to stop its production and consequently end the clinical trial agreement. No conclusions could be drawn from the study due to a small sample size being secondary to slow recruitment [25]. While favipiravir has shown efficacy against various RNA viruses, including influenza, to the extent that it was licensed for its treatment in Japan, it also demonstrated effectiveness in cell culture and mice studies against EBOV. However, it demonstrated limited protection in nonhuman primates (NHPs) and human clinical trials [2].

Galidesivir, a broad-spectrum antiviral, exhibited potent in vitro anti-viral activity against *Ebolavirus* species, specifically EBOV and SUDV. The activity of galidesivir against EBOV was confirmed in a rodent model [26]. Recent clinical trials evaluating galidesivir’s safety found it was generally well-tolerated [27]. It is yet to be evaluated in patients with EVD.

Therapeutic classes other than PIs have been attempted. Small-interfering RNA (siRNA) lipid nanoparticle products meant to interfere with viral replication, were among the first novel treatment modalities to be attempted to treat EVD patients. TKM-130803, an anti-EBOV therapeutic agent comprised of 2 siRNAs, was evaluated in a single-arm phase II trial in adults with laboratory-confirmed EVD. However, enrolment in the study stopped as the prespecified futility boundary was reached after enrolling 14 patients [28]. Another investigational product of the same class, TKM-100802 was used in the treatment of 2 patients in the US along with convalescent plasma, both of which made full recovery; however, the impact of the use of TKM-100802 could not be determined [29].

During the 2013 to 2016 outbreak in West Africa, one of the therapeutic modalities prioritized by the WHO was convalescent plasma. However, a non-randomized comparative study of 99 EVD patients in Guinea found no significant improvement in survival compared to patients treated in the same Ebola treatment unit before convalescent plasma was made available as well as those who did not have access to ABO-compatible convalescent plasma throughout the study period [30]. Although it had some limitations, as it was non-randomized, it failed to assess anti-EBOV titers and the effect of repeated administration.

## 6. What are the current means of prevention against EBOD?

While the previously discussed infection control measures remain essential for disease prevention, pre-exposure (PrEP) prevention, such as vaccines, and post-exposure prophylaxis (PEP) have been considered [31].

Four vaccines have received approval from different regulatory bodies. Ervebo, Zabdeno/Mvabea, Ad5-EBOV, and GamEvac-Combi use EBOV’s GP to elicit an immune response, with Ervebo using a recombinant vesicular stomatitis virus as vector, Zabdeno/Mvabea and Ad5-EBOV being adenovirus-based, and GamEvac-Combi being a combination of vesicular stomatitis virus (VSV) and adenovirus-based technologies [31]. Among the currently approved vaccines, only Zabdeno/Mvabea targets various species from the *Ebolavirus* genus due to its Mvabea component.

Ervebo (rVSV-EBOV) is an FDA and European Medicines Agency (EMA)-licensed, live-attenuated, replication-competent vaccine administered as a single dose. It is a genetically engineered version of the VSV, an animal virus, carrying EBOV’s GP. It has shown high immunogenicity and durability, with EBOV GP-specific antibodies persisting for up to 2 years post-vaccination [31]. Additionally, while there are a few trials assessing its safety in infants as well as in pregnant and breastfeeding women, the WHO recommends its use in active EBV outbreak areas [32]. Ervebo’s efficacy and safety, along with Zabdeno/Mvabea, was recently assessed in 1,401 pediatric (age 1 through 18) patients in a randomized clinical trial conducted by the PREVAC study group. This trial compared Zabdeno/Mvabea, Ervebo and Ervebo followed by a booster shot against a placebo group, and 30% (*n* = 407) of children were randomized into the Ervebo group and 14% (*n* = 202) were randomized to the Ervebo-booster group. A sustained antibody response was observed in both groups upon follow-up at 1 year, with the Ervebo-booster group demonstrating a slightly higher level of response rate (87% versus 93%, respectively). Only mild adverse events were reported. The FDA approved this vaccine for use in children aged 1 year and above in 2023 [33]. Ervebo was also tested in the 2015 Guinea outbreak in an open-label cluster-randomized ring vaccination trial, evaluating the efficacy of a single intramuscular dose of Ervebo in the prevention of laboratory-confirmed EVD in individuals who were in contact with a confirmed case, 10 days after randomization. Eligible contacts and contacts of contacts were randomized to either immediately receive the vaccine or receive it after 21 days. None of the immediately vaccinated contacts (2,119 out of 3,232 eligible) developed EVD at 10 days, whereas 16 cases were reported in contacts vaccinated at 21 days (2,041 out of 3,096 eligible). Ervebo proved to be 100% effective in Guinea in 2015 and later proved to be 97.5% effective in the 2018 to 2020 DRC outbreak [31,34]. Disadvantages of this vaccine are possible viral shedding secondary to its ability to replicate and the need for storage at very low temperatures (<−60°C), as it is stable for only 4 h at room temperature and 2 weeks at 2°C to 8°C, which might be challenging in LMICs [16].

Zabdeno/Mvabea requires 2 doses. Zabdeno is derived from adenovirus serotype-26 (Ad-26), and expresses EBOV’s GP, while Mvabea, a modified Vaccinia Ankara virus, expresses GPs from EBOV, SUDV, and Marburg virus. The combined vaccine approach (Ad26+MVA) has a good safety profile since it cannot replicate, is stable to store at room temperature, and lasts 6 months at 2°C to 8°C. It is multivalent after the Mvabea shot [31]. It has also been recommended by the WHO to be used in children, pregnant, and breastfeeding women in outbreak settings [32]. It has been recently tested in a randomized, placebo-controlled phase II clinical trial assessing its efficacy and safety in human immunodeficiency virus-infected adults and was found to be well tolerated and immunogenic [35]. However, it has lower efficacy compared to Ervebo and is not ideal for outbreak settings, given that 2 doses 8 weeks apart are required, and preexisting immunity to Ad-26 could theoretically affect the vaccine’s effectiveness. So far, it is only licensed by the EMA under exceptional circumstances [31].

Ad5-EBOV is a vaccine licensed by the Chinese FDA for emergency use. It is a novel recombinant adenovirus type-5 (Ad-5) vector-based vaccine. It is administered as a single dose. This vaccine has reached phase II clinical trials and proved thus far to be immunogenic and well tolerated, with only self-limiting mild adverse events reported. There is no clinical data on this vaccine concerning its durability and degree of protection. It requires storage at a temperature between 2°C and 8°C and lasts for 12 months only. One main concern around this vaccine is its use of Ad-5, a virus with preexisting antibodies against it usually present in the population, with concerns that these antibodies interfere with the ability of the vaccine to confer immunity [31].

GamEvac-Combi is a heterologous VSV and Ad-5-vectored vaccine approved for emergency use in Russia. It has recently completed phase IV clinical trials in Russia. It is administered in 2 doses, with the second dose serving as a booster, administered 21 days after the initial dose. While it boasts the advantages of both VSV and Ad5-vectored vaccines, it still carries their limitations, as concerns of decreasing immunological response due to preexisting antibodies to Ad-5 remain [31]. Data concerning its durability and threshold of protection are yet to be published.

PEP is a strategy that has shown promise against EVD. MAbs like Ebanga and Inmazeb have been used in high-risk contacts and proved to be effective as none of the contacts became symptomatic or tested positive for EBOV [36]. Interestingly, Ervebo has also shown efficacy in PEP, with none of the tested subjects infected on follow-up after 3 months [37].

However, the use of mAbs as PEP poses a problem because it could decrease the efficacy of vaccination when needed. Both the minimum time between mAbs and vaccine and the maximum time between contact and PEP administration are yet to be determined [36].

Antivirals could be an effective alternative, as favipiravir has been demonstrated to be effective in terms of prevention in a case series of 8 individuals, with no clinical or serological evidence of infection after 42 days of follow-up [38].

## 7. What are the new treatment modalities in the pipeline?

Extensive research into the EBOV genome and structure has identified various proteins essential for viral replication that can be targeted for drug development [1,2]. Several therapeutic drugs, including GP inhibitors and PIs, are currently being investigated in preclinical trials and demonstrating promising results [2]. Some of the GP inhibitor options, identified through computational analysis, are FDA-approved drugs like clomiphene that has demonstrated efficacy in cell cultures only [2]. Advances in artificial intelligence and machine learning could prove indispensable for categorizing compounds identified through high-throughput screening of chemical data as potential treatment options against ebolaviruses [39].

Recently, host factors involved in viral processes and immune response have garnered attention as therapy targets. Amiodarone, shown to inhibit viral membrane fusion in vitro, was tested in Sierra Leone but later withdrawn due to inconclusive effects in animal models [40]. Another potential interaction of interest is the interaction between ebolaviruses and the Niemann–Pick C1 receptor (NPC-1), with multiple molecules exhibiting adequate anti-Ebola activity in vitro, such as MBX2254 and MBX2270 [41], and the newly discovered 2 diaryl sulfide derivatives, SC198 and SC073, and SC816 [42]. In view of this interaction’s importance, a bispecific-antibody that targets both EBOV’s GP and NPC-1 has been devised and demonstrated efficacy in murine models [43].

Combination therapies which include FDA-approved medications (toremifene-mefloquine-posaconazole, toremifene-clarithromycin-posaconazole) have emerged to potentially prevent resistance and synergistically control infection, lowering drug dosing and toxicity [2]. These proposed combinations have been shown to inhibit various stages of viral entry and replication in in vitro studies [2]. One of the benefits of such combinations is that they are already well studied with known side effects and drug–drug interactions that are already well characterized.

Adaptor-associated kinase 1 (AAK1) inhibitors have surfaced as promising therapeutic agents against multiple viruses, including ebolaviruses. This is mainly due to their ability to inhibit the clathrin-mediated pathway, essential for viral entry into host cells. An example of a molecule of this class that exhibited anti-EBOV activity in vitro is Sunitinib in combination with Erlotinib [44].

Given the documented efficacy of mAbs in the fight against EVD, with the only FDA-approved therapeutics thus far belonging to this class of therapy, ongoing efforts are in place to devise mAbs that are cross-neutralizing and cross-protective among different *Ebolavirus* species, especially in light of SUDV re-surfacing as an outbreak etiology after 10 years of no reports. While multiple broad-spectrum mAbs are currently being assessed in NHPs with promising results, a cocktail of 2 mAbs isolated by Bornholdt and colleagues MBP134AF seems to be promising. This mAb cocktail successfully protected ferrets and NHPs against EBOV, SUDV, and Bundibugyo species [45]. It is currently in clinical trials as a single or combination therapy with remdesivir for the treatment of EBOD secondary to SUDV in Uganda [46,47]

## Conclusion

Significant progress has been made in EVD treatment and prevention, but many questions remain unanswered. mAbs have notably reduced EVD mortality, even in critical cases, yet mortality remains high at 30% [22] with secreted GP as a potential culprit, as one proposed mechanism is this protein acting as a decoy to these monoclonal antibodies [48]. While existing therapies only target EBOV, the development of pan-species mAbs is crucial so that other species are not overlooked [2]. Furthermore, the selection for viral escape variants for immunotherapeutics targeting a single epitope remains a concern, with a cocktail of antibodies targeting various epitopes offering a compelling solution [49]. Regarding prevention, further studies are needed to investigate the duration of vaccine-generated immunity, and the safety of vaccines in immunocompromised patients. Further exploration of PIs as a means of PEP is appealing given their promise in a case series [38].
